# Métastase géante sous cutanée d'un carcinome vésiculaire de la thyroïde: à propos d'un cas

**DOI:** 10.11604/pamj.2018.31.245.9607

**Published:** 2018-12-26

**Authors:** Ny Ony Tiana Andrianandrasana, Malala Razakanaivo, Marie Ida Rahantamalala, Florine Josoa Rafaramino

**Affiliations:** 1 Service Oncologie-Radiothérapie, Hôpital Joseph Ravoahangy Andrianavalona CHU Antananarivo, Madagascar; 2 Service de Médecine Interne CHU Ambohidratrimo, Anosiala, Madagascar

**Keywords:** Cancer thyroïde, métastase, cuir chevelu, Thyroid cancer, metastasis, scalp

## Abstract

La localisation secondaire sous-cutanée de carcinome vésiculaire de la thyroïde est inhabituelle. Elle représente au plus 5,8% de sites métastatiques préférentielles de la thyroïde. Il s'agit d'une femme de 60 ans, présentant une énorme tuméfaction hypervasculaire du tissu sous-cutané du crâne, d'évolution lente, après 7 ans d'une lobectomie droite de la thyroïde. Le bilan anatomo-pathologique a conduit au diagnostic de carcinome vésiculaire de la thyroïde. Une intervention chirurgicale consistait à réduire la lésion du crâne ainsi qu'une thyroïdectomie a été réalisée. Actuellement, elle est en attente d'un traitement par l'iode radio-actif. La métastase de carcinome vésiculaire du scalp existe malgré que c'est rare. Toutefois, à ce stade le pronostic reste défavorable.

## Abstract (en)

Secondary subcutaneous metastasis originating from vesicular thyroid carcinoma is unusual. It accounts for more than 5.8% of most common sites of metastasis for thyroid carcinoma. We here report the case of a 60-year old woman presenting with slowly evolving giant hypervascular lesion in the subcutaneous tissue of the skull occurred 7 years after right thyroid lobectomy. Anatomopathological examination enabled the diagnosis of vesicular thyroid carcinoma. Surgery was based on reduction of the skull lesion as well as on thyroidectomy. Currently, the patient is waiting for radioactive iodine treatment. Metastasis from vesicular carcinoma of the scalp, even though rare, can occur. However, this stage is associated with unfavorable prognosis.

## Introduction

Le cancer de la thyroïde représente 1 à 2% des maladies néoplasiques et 90% des cancers endocriniens [1]. Le type vésiculaire est le plus courant des carcinomes thyroïdiens après le type papillaire. Il produit le plus de métastase mais rarement au niveau de tissu sous-cutané. Toutefois, il est considéré de bon pronostic [2]. L´objectif est de rapporter une forme inhabituelle de localisation secondaire de carcinome vésiculaire et de discuter de l´attitude thérapeutique.

## Patient et observation

Une femme de 60 ans, cultivatrice, domiciliée à 600km du centre de référence, qui était adressée pour une suite de prise en charge d'une tumeur fronto-pariéto-occipitale gauche, négligée, évoluant depuis novembre 2010, de façon progressive. L'apparition d'une douleur locale associée à une altération de l'état général en décembre 2013 motivait la patiente à se faire consulter dans un centre hospitalier. Une biopsie de la tumeur était réalisée, révélant le diagnostic d'une métastase de carcinome vésiculaire de la thyroïde.

**Figure 1:**
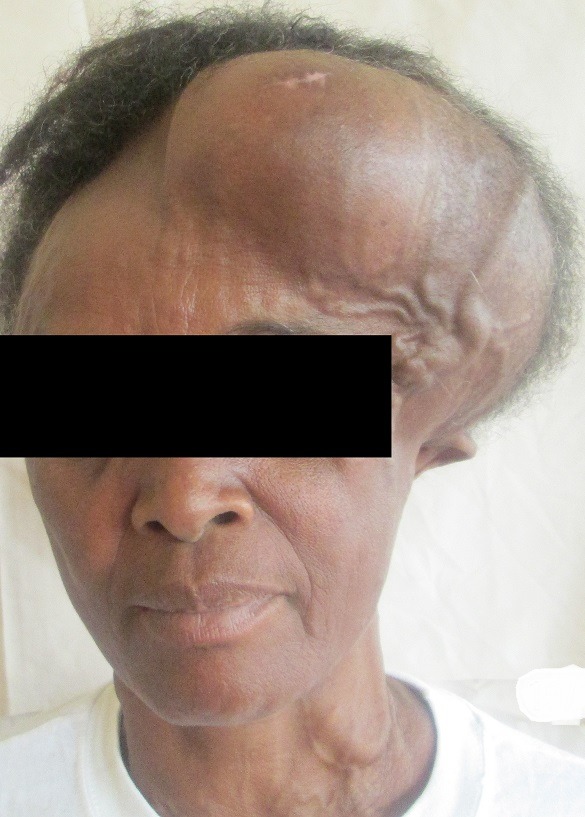
métastase de carcinome vésiculaire de la thyroïde fronto-pariéto-occipitale gauche

**Figure 2:**
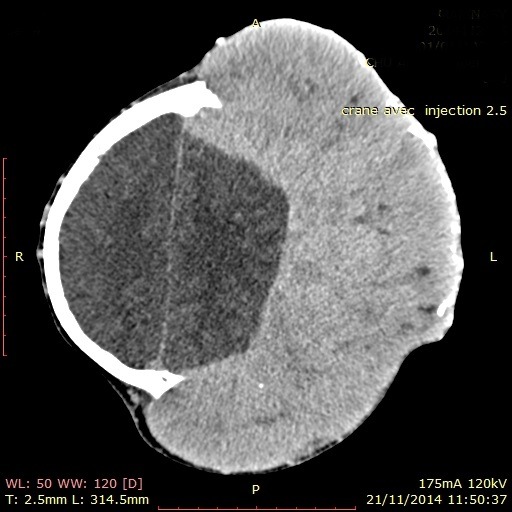
une masse fronto-pariéto-occipitale gauche avec un début d'infiltration parenchymateuse corticale gauche et une lyse osseuse

**Figure 3:**
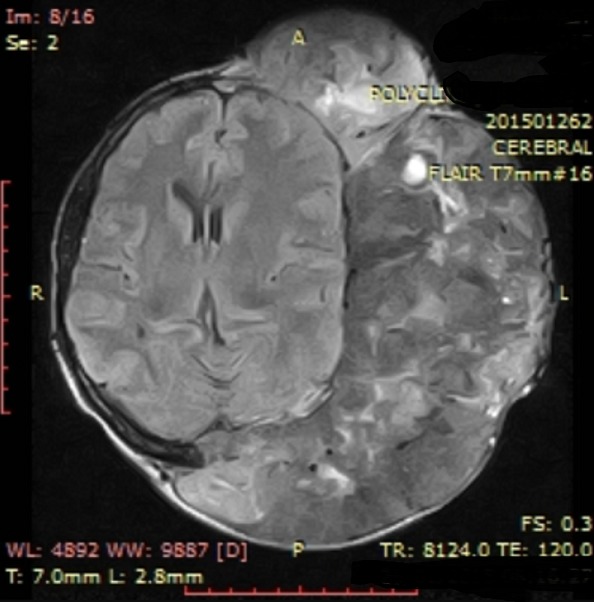
hypervascularisation au sein d'une métastase de carcinome vésiculaire de la thyroïde

Dans ses antécédents, on notait une notion de lobectomie gauche de la thyroïde en 2008, sans recherche histologique. L'examen clinique montrait une augmentation du volume de la thyroïde du côté droit, avec présence de nodule unique, une volumineuse masse fronto-pariéto-occipitale gauche, très hypervascularisée, rénitente, peu sensible à la palpation, mesurant 22cm x 17cm (Figure 1). Les examens biologiques étaient sans particularité. Elle était en euthyroidie. L'échographie thyroïdienne objectivait un goitre lobaire droit hétérogène nodulaire. La radiographie du thorax montrait une image nodulaire basale droite faisant évoquer un lâcher de ballons. Le scanner cérébral retrouvait une masse fronto-pariéto-occipitale gauche avec un début d'infiltration parenchymateuse corticale gauche et une lyse osseuse (Figure 2). Une angiographie par résonance magnétique était demandée pour évaluer l'hypervascularisation en vue d'une chirurgie de réduction tumorale (Figure 3).

Une chirurgie de type réduction tumorale au niveau du scalp et thyroïdectomie totale suivie d'irathérapie étaient retenus en réunion de concertation pluridisciplinaire. La thyroïdectomie se déroulait sans difficulté pourtant un saignement important s'était produit dès l'incision du scalp, empêchant ainsi la réduction tumorale.

## Discussion

Le carcinome vésiculaire est le sous type de cancer de la thyroïde qui est considéré comme le plus agressif, caractérisé par l´invasion vasculaire, expliquant la fréquence de la localisation à distance [1, 2]. Il s´agit du premier cas observé au sein de notre service, sur 7 à 8 cas de carcinome thyroïdien recensé par an [3]. La manifestation clinique est typique de localisation sous-cutanée du scalp, marquée par une évolution lente. La prise en charge thérapeutique est très limitée par l'énorme taille tumorale, la vascularisation importante et le faible pouvoir d´achat de notre population surtout par rapport au coût élevé du produit radioactif iodé.

Les tumeurs métastatiques du cuir chevelu sont habituellement observées à partir des tumeurs malignes du poumon, du sein, de la prostate et relativement rare de cancers de la thyroïde. L'incidence de cette dernière est rapportée entre 2,5 % et 5,8 % des cas de métastases des carcinomes vésiculaires de la thyroïde. En outre, parmi les localisations sous-cutanées rapportées dans la littérature telles que l'abdomen, le dos, le bassin et la cuisse, le cuir chevelu et le cou sont décrits comme les zones les plus fréquemment touchées [4]. La métastase de cuir chevelu se présente habituellement comme un gonflement sur une bonne région scapulaire, peu symptomatique, avec presque exclusivement lytique, très hypervascularisée, identique à notre cas.

Certains auteurs ont fait valoir que les patients ont généralement une longue évolution clinique avant le diagnostic de lésion du crâne [5], situation prouvée par Negamine *et al.* dans son étude rapportant 12 cas de métastase sous-cutanée de carcinome vésiculaire, qui ont signalé une durée moyenne de 23,3 ans, du diagnostic de tumeur de la thyroïde jusqu´à la découverte de la métastase du crâne [6], largement supérieur à notre cas, qui avait duré 7 ans. Le traitement des patients présentant des métastases inclus une thyroïdectomie totale, l´excision de la lésion autant que possible et l'administration de la thérapie à l´iode radioactif [7]. Certains auteurs soutiennent l´utilisation de la radiothérapie externe à la fois pour contrôle locorégional et contrôle des métastases inopérables [8, 9].

Le meilleur traitement pour les métastases du crâne, aussi étendue comme chez notre patiente, reste à déterminer, par la difficulté du geste chirurgical, en raison d'une large participation du crâne radiographiquement démontrée et par la présence d´une hypervascularisation. Cependant, plus il reste un important résidu tumoral, plus l'efficacité de thérapie par l'iode radioactif est moindre et plus le pronostic est mauvais [10].

## Conclusion

En conclusion, bien que rare, la métastase de carcinome vésiculaire sous-cutanée doit être suspectée devant toute tuméfaction du crâne. Et nous avons rappelé que, le délai de diagnostic long avec pour corollaire une prise en charge tardive comme le cas de notre patiente, a contribué à la mauvaise issue de cette pathologie grave dans les pays en développement. Même si la réduction tumorale est recommandée dans les cancers de la thyroïde métastatique, elle est très difficile au niveau du scalp à cause de l'hypervascularisation.

## Conflits d’intérêts

Les auteurs ne déclarent aucun conflit d'intérêts.

## Remerciements

Tous les auteurs adressent les plus sincères remerciements à toutes personnes physiques ou morales qui ont contribué à la réalisation de ce travail.
